# Y-chromosome DNA Is Present in the Blood of Female Dogs Suggesting the Presence of Fetal Microchimerism

**DOI:** 10.1371/journal.pone.0068114

**Published:** 2013-07-08

**Authors:** Sandra M. Axiak-Bechtel, Senthil R. Kumar, Sarah A. Hansen, Jeffrey N. Bryan

**Affiliations:** 1 Comparative Oncology and Epigenetics Laboratory, Department of Veterinary Medicine and Surgery, University of Missouri, Columbia, Missouri, United States of America; 2 Harry S. Truman Veterans Hospital, Columbia, Missouri, United States of America; Baylor College of Medicine, United States of America

## Abstract

Fetal microchimerism has been suggested to play contradictory roles in women’s health, with factors including age of the recipient, time elapsed since microchimerism occurred, and microchimeric cell type modulating disease. Both beneficial and harmful effects have been identified in wound healing and tissue regeneration, immune mediated disease, and cancer. This area of research is relatively new, and hindered by the time course from occurrence of fetal microchimerism to the multi-factorial development of disease. Dogs represent an excellent model for study of fetal microchimerism, as they share our environment, have a naturally condensed lifespan, and spontaneously develop immune-mediated diseases and cancers similar to their human counterparts. However, fetal microchimerism has not been described in dogs. These experiments sought preliminary evidence that dogs develop fetal microchimerism following pregnancy. We hypothesized that Y chromosomal DNA would be detected in the peripheral blood mononuclear cells of female dogs collected within two months of parturition. We further hypothesized that Y chromosomal DNA would be detected in banked whole blood DNA samples from parous female Golden Retrievers with at least one male puppy in a prior litter. Amplification of DNA extracted from five female Golden Retrievers that had whelped within the two months prior to collection revealed strong positive bands for the Y chromosome. Of banked, parous samples, 36% yielded positive bands for the Y chromosome. This is the first report of persistent Y chromosomal DNA in post-partum female dogs and these results suggest that fetal microchimerism occurs in the canine species. Evaluation of the contributions of fetal microchimeric cells to disease processes in dogs as a model for human disease is warranted.

## Introduction

Microchimerism is the co-existence of two cell populations originating from two different persons in the same individual, caused by the transfer of a low number of cells from one person to another in certain circumstances [1]. This can result from blood transfusion, organ transplantation, hematopoietic stem cell transplantation, fraternal twin gestation, or most commonly, pregnancy [1]–[3]. Fetal microchimerism (FMC) is the persistence of fetal cells in maternal organs and circulation as a result of pregnancy. Fetal microchimerism has been reported to have overlapping or even conflicting roles in disease processes. For example, the persistence of fetal cells in maternal organs may lead to a graft versus host reaction, resulting in chronic inflammation, tissue damage, and immune-mediated disease [4], [5]. Alternatively, these fetal cells have been shown to differentiate into functional immune cells, participate in tissue repair, or provide tumor surveillance. They are thought to have a role in preventing cancer as well as both causing and modulating autoimmune disease [6]–[10]. Unraveling the function of these microchimeric cells is imperative to provide insight into their role in health and disease. Currently, mouse models have identified benefits of induced FMC in chronic disease risk [11]. A naturally occurring, large animal model with risk for spontaneous diseases that frequently affect humans and which have a high frequency of FMC will allow hypothesis testing in a more realistic setting.

Fetal microchimerism is detected in women that have had at least one son by detecting DNA from the Y chromosome. This male chromosomal DNA can be detected within total DNA extracted from circulating peripheral blood mononuclear cells or from whole blood amplified using polymerase chain reaction (PCR) assays. Using these techniques, FMC has been identified following pregnancy in mice, rats, cows, and humans [12]–[18]. Studies are ongoing in women to further elucidate the role of FMC in disease processes. However, these are hindered by the long and challenging process of collecting epidemiologic data on multi-factorial diseases and a lack of repository of blood for research. Companion dogs develop numerous immune-mediated diseases and cancers with etiologies and clinical courses similar to those seen in humans [19]–[23]. The condensed lifespan of dogs make them ideal to model the association of FMC with development of these diseases. Although microchimerism has been described in dogs following organ transplantation and hematopoietic reconstitution, it has not been described in dogs following pregnancy [24], [25].

The objective of our study was to determine if Golden Retrievers develop fetal microchimerism following pregnancy. We hypothesized that Y chromosomal DNA would be detected in the peripheral blood mononuclear cells of female dogs whelping male puppies collected within two months of parturition. We further hypothesized that Y chromosomal DNA would be detected in banked whole blood DNA samples from parous Golden Retrievers with at least one male puppy in a prior litter. Confirmation of these hypotheses would support the existence of FMC in dogs. Demonstration of evidence supporting FMC in companion dogs would underpin future research in the mechanisms, selection, persistence, and health effects of FMC. Our results clearly demonstrate the presence of Y chromosomal DNA in female dogs in both the newly-parous and late post-parturient period, suggesting the existence and persistence of FMC in dogs.

## Materials and Methods

### Collection of Blood Samples and Processing

This protocol was carried out in strict accordance with and approval of the University of Missouri Animal Care and Use Committee (Protocols 7422 and 7360), with informed dog-owner consent. To determine if post-natal FMC occurs in dogs, blood samples were acquired from five female parous dogs within two months of parturition in BD Vacutainer CPT Cell Preparation Tubes with Sodium Citrate anticoagulant with Ficoll-HyPaque media within the tube by collecting veterinarians, with the assistance of the Golden Retriever Club of America. Samples were spun at 1500 g for 30 min to separate mononuclear cells, according to manufacturer instructions, and then shipped on ice to the University of Missouri Comparative Oncology Laboratory. The PBMCs were collected and diluted in phosphate buffered saline (PBS). Genomic DNA was isolated from the cell pellet as described below.

Banked sample DNA was extracted as described below from whole blood digestion and stored for various periods of time. To determine if persistent FMC occurs in dogs, whole blood DNA samples were acquired from the Orthopedic Foundation for Animals (Columbia, MO) from 100 parous golden retrievers selected based on an e-mail survey seeking dogs which had a history of giving birth to litters containing at least one male offspring. Dogs with a history of blood transfusion, a theoretical source of microchimerism, were excluded through the survey. For positive and negative controls, the whole blood sample (∼ 2 ml in heparinized vacutainer tubes) or PBMCs were collected from male and nulliparous female dogs, respectively.

### Genomic DNA Isolation

DNA was isolated from the dog blood samples or PBMCs using DNeasy Blood and Tissue Kit (Qiagen, Santa Clarita, CA) according to manufacturer’s instructions. DNA concentration was determined using Nanodrop 1000 Spectrophotometer (Thermo Scientific, Wilmington, DE).

### PCR Assay for Microchimerism

The microchimerism PCR assay was performed as follows: Each DNA preparation (50 ng of male control sample and 100 ng of all female DNA samples) was subjected to a first round of PCR using canine male chromosome (Y) specific primers, followed by a nested amplification of the initial PCR product (50 ng of male control sample was used due to over-amplification of the male control). The primers were designed based on the published sequences of canine male-specific DNA fragments originally detected in Labrador Retrievers [26]. Two sets of primers were synthesized; one set of primers for a 650 bp fragment, and a second set of nested primers within the 650 DNA fragment (∼320 bp fragment). In each PCR run, male and nulliparous female dog DNA was used as positive and negative controls, respectively, with a water control used to detect contamination. The DNA samples from the dogs were subjected to first round of PCR using primers to amplify the 650 bp fragment (native). Typical reactions included HF Phusion buffer (pH 8.0) containing MgCl_2_, dNTP’s, and Phusion DNA polymerase (Thermo Scientific, Rockford, IL). The PCR conditions were as follows: denaturation at 98°C for 30 s, followed by 28 cycles at 98°C for 20 s, 65°C for 30 s, and then 72°C for 30 s, with a final extension at 72°C for 8 min. For the nested primers, approximately 0.25 µl of the primary reaction sample was used as a template with the internal nested set of primers (320 bp fragment) and further amplified for an additional 10 cycles. In order to visualize the Y-specific bands, an aliquot from each PCR reaction was electrophoresed in a 1% agarose gel in Tris-borate containing Gel-Red (Biotium, Hayward, CA). Gels were photographed on a UV-trans-illuminator (BioDoc-UVA Imaging System, Upland, CA) and bands visualized with ImageJ (http://imagej.nih.gove/ij).

To test the detection sensitivity of the PCR, the male and nulliparous female blood samples were serially diluted in different ratios (male-to-female-1∶1 to 1∶90,000) and genomic DNA was isolated from the mixed samples. The first round of PCR was performed with native primers as mentioned above followed by a second round of PCR using nested primers. The PCR products were visualized using gel electrophoresis as mentioned above.

Gel analysis was performed using ImageJ. Regions of interest were drawn around the region of 320 bp and 1900 bp in each lane and the intensities measured. A ratio of band region to lane background was calculated using the following formula: relative intensity = i_320_/i_1900_. Negative control lanes were averaged and the standard deviation calculated. Intensities greater than the mean plus 2 standard deviations were considered to be positive lanes. The positive control bands were significantly more intense than the corresponding regions in the negative control lanes (P<0.001) and all were much greater than the calculated cutoff for positivity. True negative female DNA and water lanes yielded no bands.

### Statistical Analysis

Mann-Whitney Rank Sum Test was used to compare groups for difference in relative intensity. Linear correlation was used to compare band intensity to time between previous parturition and blood collection. All evaluations were performed using Sigma-Stat (Systat Software Inc. Chicago, IL). P values <0.05 were considered significant.

## Results

The newly parous females were five Golden Retrievers that had whelped a litter containing male puppies in the prior two months. The banked sample population included female Golden Retrievers of varying ages, and the blood collection ranging between a few months to several years after first delivery of their litter.

**Figure 1:**
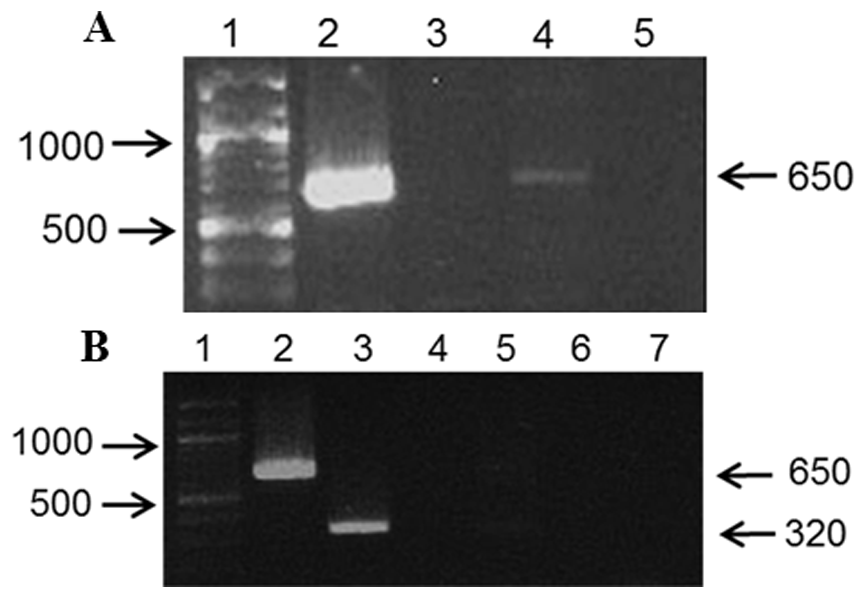
PCR reactions for positive and negative controls. Figure 1A represents the native primer for dog Y-specific DNA fragment of 650 bp, and Figure 1B represents the nested primer or ∼320 bp within the 650 bp fragment.

We optimized the nested PCR assay to increase the sensitivity of our detection and decrease the risk of mispriming with the native primer alone. The outer primer is complementary to dog Y-specific DNA (∼650 bp) and the nested primer (∼320 bp) was designed to be complementary within the 650 bp fragment. The primer sequences are presented in Table 1. The PCR reactions were robust and repeatable (Figure 1A, 1B). DNA from nulliparous females used as a control consistently lacked the target amplicons, confirming the assay specificity for the Y-chromosome.

**Table 1:** PCR primers used for FMC.

PCR	
Native	F: 5′-GTC CTG GGT TCG GGT TAG TGT TAG-3′
	R: 5′-GTC CTG GGT TGA AGC CCT ACA TTG-3′
Nested	F: 5′-AAG CCC TAC ATT GGG ATC TCT GCT-3′
	R: 5′-TGA CTC AGT TGC CTC ATC ACA GGA-3′
F-Forward; R-Reverse	

**Figure 2:**
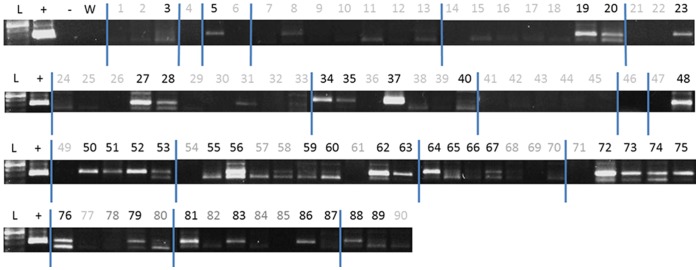
Presence of 320 bp segments of Y-chromosomal DNA following nested PCR amplification performed on banked female Golden Retriever whole-blood DNA samples. Initial PCR was performed with 650 bp amplicon primers followed by 320 bp nested primer amplification. The PCR products were electrophoresed in a 1% agarose gel with Tri-borate containing Gel-Red and visualized with Bio-Doc-UVA Imaging System. (L) = 100 bp DNA ladder; (+) = male DNA positive control; (−) = female nulliparous DNA negative control; (W) = water template control; (black) numbers = female samples positive for the presence of 320 bp Y-chromosome DNA segments; (gray) numbers = female samples negative for the presence of 320 bp Y-chromosome DNA segments.

**Figure 3:**
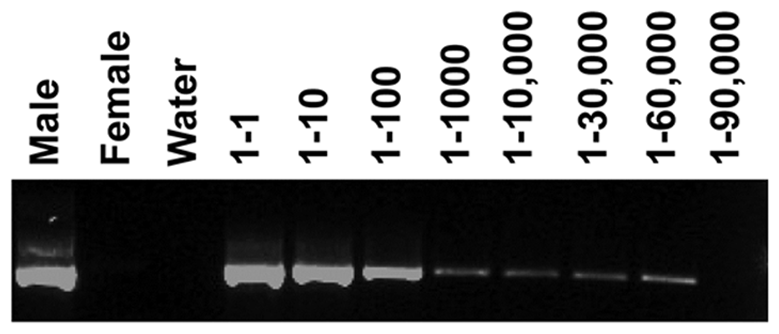
Nested PCR of dilutions of male: female blood, with male to female ratios of 1∶1 to 1∶90,000. Bands of Y chromosomal DNA are detected in as small as 1∶60,000-fold M:F diluted samples, but not in 1∶90,000-fold diluted samples.

Amplification of DNA extracted from all five dogs that had whelped within the two months prior to collection uniformly revealed strong positive bands for the Y chromosome (Supplemental data). Ninety banked samples from female Golden Retrievers were tested and 38 samples were positive. However, on further owner questioning, 9 of these 38 positive samples were from dogs which had only whelped litters after blood collection for banking. All nine of these female dogs had male littermates. Therefore, of 81 banked blood DNA samples collected from confirmed parous Golden Retrievers, 29 (36%) yielded positive bands for the Y chromosome. A composite gel for the samples is shown in Figure 2. The relative intensities of the bands are presented in Table 2. From time of whelping to time of banked sample collection varied from three months to eight years. There was no correlation between time from last pregnancy and band intensity. The sensitivity of PCR was also determined. Using nested PCR, bands of Y chromosomal DNA could be detected in 1∶60,000-fold M:F diluted samples, but not in 1∶90,000-fold diluted samples. (Figure 3).

**Table 2:** Dog Characteristics and Gel Analysis.

**Animal Number**			**1**	**2**	**3**	**4**	**5**	**6**	**7**	**8**	**9**	**10**	**11**	**12**	**13**	**14**	**15**	**16**	**17**	**18**	**19**	**20**	**21**	**22**	**23**
**Result**					**+**		**+**														**+**	**+**			**+**
**Intensity**			1.070	1.098	1.291	1.039	1.378	0.995	1.185	0.969	0.963	1.023	1.017	1.067	0.925	0.997	1.034	1.052	1.088	0.980	2.182	1.667	1.066	1.051	1.455
**Months Since Parturition**					75	30	22	45		14	45			47			N	73	N	14	16	55		65	96
**Male sibling if no litters prior**																Y			Y						
**Animal Number**	**24**	**25**	**26**	**27**	**28**	**29**	**30**	**31**	**32**	**33**	**34**	**35**	**36**	**37**	**38**	**39**	**40**	**41**	**42**	**43**	**44**	**45**	**46**	**47**	**48**
**Result**				**+**	**+**						**+**	**+**		**+**			**+**								**+**
**Intensity**	1.149	1.013	0.987	2.147	1.504	0.988	0.998	1.169	1.049	1.085	1.988	1.371	1.065	2.708	1.123	1.090	1.264	1.006	1.030	0.997	1.018	0.962	0.974	0.947	2.957
**Months Since Parturition**	6	14	66	3	3			9	41	26	59	18	55	P	5	36	7			3				18	18
**Male sibling if no litters prior**																									
**Animal Number**	**49**	**50**	**51**	**52**	**53**	**54**	**55**	**56**	**57**	**58**	**59**	**60**	**61**	**62**	**63**	**64**	**65**	**66**	**67**	**68**	**69**	**70**	**71**	**72**	**73**
**Result**		**+**	**+**	**+**	**+**		**+**	**+**			**+**	**+**		**+**	**+**	**+**	**+**	**+**	**+**					**+**	**+**
**Intensity**	1.067	2.116	1.766	2.434	1.448	0.820	1.399	2.686	1.156	1.121	1.323	1.273	0.745	2.631	1.981	3.199	1.756	1.337	1.640	1.183	0.817	1.168	0.731	2.947	2.191
**Months Since Parturition**	25	36	N	37	N	67		N	57	7	17	9			26	31	15	N	23			N		N	40
**Male sibling if no litters prior**			Y		Y			Y										Y				Y		Y	
**Animal Number**	**74**	**75**	**76**	**77**	**78**	**79**	**80**	**81**	**82**	**83**	**84**	**85**	**86**	**87**	**88**	**89**	**90**								
**Result**	**+**	**+**	**+**			**+**		**+**		**+**			**+**	**+**	**+**	**+**									
**Intensity**	1.933	2.217	2.053	1.070	1.093	1.563	1.113	2.710	1.184	1.892	1.175	1.039	1.919	1.246	1.714	1.274	1.122								
**Months Since Parturition**	N	23		18		N		N		19		10	36	4	46										
**Male sibling if no litters prior**	Y					Y		Y																	

Animal number: Corresponds to the gel lanes in Figure 2; Result: ‘+’ denotes FMC positive; blank denotes negative; Intensity: relative intensity of Y chromosomal band to background gel; Months since parturition: duration of time passed since last litter containing male offspring to date of blood draw; Male sibling if no litters prior: presence of male sibling in birth litter if nulliparous at time of blood draw.

## Discussion

Microchimerism was detectable within two months post-whelping in all post-natal dog samples acquired. Persistent Y chromosomal DNA was found in banked samples in 29 of 81 dogs with at least one litter and one male puppy as late as 96 months post-parturition. Y chromosomal DNA detection was sensitive, and bands were detected at 1∶60,000 fold M:F dilutions by PCR, suggesting that as few as one male cell in 60,000 female cells could yield a positive result. These results indicate persistent fetal microchimerism in dogs. The actual rate of microchimerism may be higher, as the assay performed here would not detect female microchimerism.

Dogs, cows, and humans have different types of placentation. Cows have epitheliochorial placentation, with no trophoblast cell invasion beyond the uterine epithelium. Dogs have endotheliochorial placentation, with the uterine epithelium breached and trophoblastic cells in direct contact with endothelial cells of maternal uterine blood vessels. Women have hemochorial placentation, with maternal uterine blood vessels infiltrated by trophoblast cells causing rupture and release of blood into the intervillous space. Despite the difference in the type of placentation and the lack of intimacy between vasculature of the uterus and placenta, fetal microchimerism is still found in the cow [12], [13], [27]. The dog has more intimate connections between uterine and fetal vasculature than the cow, therefore, type of placentation is not expected to affect the occurrence of fetal microchimerism.

An email survey of dog owners was used to confirm whelping, parturition date, and sex of puppies. Although recall bias may have occurred when using an email survey, we limited our dogs to purebred Golden Retrievers whose owners participate in the Golden Retriever Club of America. These pet owners keep detailed records on number of pregnancies, parturitions, and sex of puppies for the purposes of pure-bred puppy certification. This record keeping allowed minimization of recall bias.

Retrospectively, nine of the dogs with banked blood samples and a positive result were discovered to be nulliparous at the time of sample collection. The known sources of microchimerism in the dog, organ transplantation and blood transfusion, were ruled out using an owner survey prior to sample analysis. Interestingly, approximately 20% of women with no history of a male birth have male microchimerism in their peripheral blood. Postulated explanations of this phenomenon include early absorbed pregnancy, an unborn male twin, or older male sibling microchimeric cells transferred by maternal circulation to the fetus [28]. All nine nulliparous dogs with circulating Y chromosomal DNA, without a history of whelping, had male littermates. Therefore, *in utero* sibling microchimerism is a likely mechanism for the Y chromosomal DNA found in these dogs, although other mechanisms cannot be excluded. Given the 100% positivity of dogs with confirmed male offspring within two months post-partum, and 36% of dogs with confirmed male offspring in banked samples, our results strongly support true and persistent fetal microchimerism in the dog.

Detection of the Y chromosome in females with a history of giving birth to male progeny is the most straightforward way to detect and study fetal microchimerism, and was elected as the method of choice in this investigation. More detailed methods in future studies will be undertaken to determine the true cell of origin (fetal versus littermate), female fetal microchimerism, and phenotype of circulating cells.

Companion dogs develop many diseases similar to their human counterpart, and combined with a shared environment and readily available banked blood samples, dogs are an ideal model for human diseases. Furthermore, epidemiologic data collection and interpretation can be obtained much more efficiently due to the condensed lifespan. Immune-mediated disease is common in the pet dog population, including lymphocytic thyroiditis, which parallels Hashimoto’s thyroiditis, and systemic lupus erythematosus [20], [21], [29]. Both of these diseases are suspected to be caused, in part, or exacerbated by FMC in women following pregnancy [30]. On the other hand, FMC is thought to have a protective role in cancer formation and prevention. Women with children have a decreased risk of and then a better prognosis for breast cancer and lung cancer, both of which occur spontaneously in dogs [7], [8], [31], [32]. Lymphoma, a systemic immunologic cancer, occurs less commonly in women and intact female dogs compared to men and male dogs or neutered female dogs, respectively [33]. Furthermore, FMC cells may participate in wound healing and may enhance wound healing and tissue regeneration in cases of maternal deficiency [5], [10], [34], [35]. Given these conflicting roles of FMC in women and murine models, it is imperative to define the role of FMC in various disease processes prior to consideration of using FMC in therapy. Many dogs have a traceable lineage, share environmental exposures with humans, and develop multi-factorial diseases which recapitulate their human counterparts [22], [23]. Straightforward sample collection and similar medical monitoring capabilities in veterinary medicine make companion dogs a very attractive model for further study.

## Conclusion

This study demonstrates that persistent male microchimerism of probable fetal origin occurs in the pet dog population. There is evidence in women that FMC may have conflicting roles in disease formation; including the triggering of autoimmune disease as well as protection against cancer and participation in wound healing. The dog represents an excellent model of many ailments in people, and the presence of FMC in dogs will allow studies which further elucidate its role in health and disease.

## Supporting Information

Figure S1 **Gel electrophoresis image showing bands present following amplification using nested primers for 10 rounds of PCR on previously amplified PCR products.** Lane 1: ladder, 2: male positive control, 3: female negative control, 4: water control, 5:#4, 6:#5, 7:#21, 8:#22, 9:#23.
(JPG)

Figure S2 **Gel electrophoresis image showing bands present following amplification using nested primers for 10 rounds of PCR on previously amplified PCR products.** Top Half: Lane 1: ladder, 2: male positive control, 3: female negative control, 4: water control, 5:#1, 6:#2, 7:#3, 8:#5, 9:#6. Bottom Half: Lane 1: ladder, 2:#7, 3:#8, 4:#9, 5:#10, 6:#11, 7:#12, 8:#13, 9:#20.
(JPG)

Figure S3 **Gel electrophoresis image showing bands present following amplification using nested primers for 10 rounds of PCR on previously amplified PCR products.** Top Half: Lane 1:ladder, 2:male positive control, 3:female negative control, 4:water control, 5:#14, 6:#15, 7:#16, 8:#17, 9:#18, 10:#19, 11:#20. Bottom Half: Lane 1:ladder, 2:#24, 3:#25, 4:#26, 5:#27, 6:#28, 7:#29, 8:#30, 9:#31, 10:#32, 11:#33.
(JPG)

Figure S4 **Gel electrophoresis image showing bands present following amplification using nested primers for 10 rounds of PCR on previously amplified PCR products.** Top Half: Lane 1:ladder, 2:male positive control, 3:female negative control, 4:water control, 5:#34, 6:#35, 7:#36, 8:#37, 9:#38, 10:#39, 11:#40. Bottom Half: Lane 1: ladder, 2:male positive control, 3:#41, 4:#42, 5:#43, 6:#44, 7:#45.
(JPG)

Figure S5 **Gel electrophoresis image showing bands present following amplification using nested primers for 10 rounds of PCR on previously amplified PCR products.** Top Half: Lane 1:ladder, 2:male positive control, 3:female negative control, 4:water control, 5:#47, 6:#48, 7:#49, 8:#50, 9:#51, 10:#52, 11:#53. Bottom Half: Lane 1:ladder, 2:#54, 3:#55, 4:#56, 5:#57, 6:#58, 7:#59, 8:#60, 9:#61, 10:#62, 11:#63.
(JPG)

Figure S6 **Gel electrophoresis image showing bands present following amplification using nested primers for 10 rounds of PCR on previously amplified PCR products.** Top Half: Lane 1: ladder, 2:male positive control, 3:female negative control, 4:water control, 5:#64, 6:#65, 7:#66, 8:#67, 9:#68, 10:#69, 11:#70. Bottom Half: Lane 1: ladder, 2:#71, 3:#72, 4:#73, 5:#74, 6:#75, 7:#76, 8:#77, 9:#78, 10:#79, 11:#80.
(JPG)

Figure S7 **Gel electrophoresis image showing bands present following amplification using nested primers for 10 rounds of PCR on previously amplified PCR products.** Top Half: Lane 1: ladder, 2:male positive control, 3:female negative control, 4:water control, 5:#81, 6:#82, 7:#83, 8:#84, 9:#85, 10:#86, 11:#87. Bottom Half: Lane 1: ladder, 2:male positive control, 3:#88, 4:#89, 5:#90, 6:blank, 7:blank.
(JPG)
