# Supplementary figures and images for: Y-chromosome DNA Is Present in the Blood of Female Dogs Suggesting the Presence of Fetal Microchimerism
Source: PLoS One. 2013 Jul 8;8(7):e68114. doi: 10.1371/journal.pone.0068114 (PMC3704588; doi:10.1371/journal.pone.0068114)

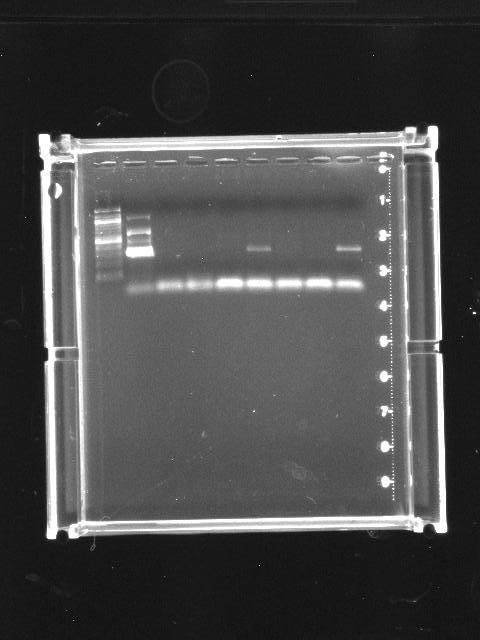

Supplement: Figure S1 — Gel electrophoresis image showing bands present following amplification using nested primers for 10 rounds of PCR on previously amplified PCR products. Lane 1: ladder, 2: male positive control, 3: female negative control, 4: water control, 5:#4, 6:#5, 7:#21, 8:#22, 9:#23. (JPG) [file pone.0068114.s001.jpg]

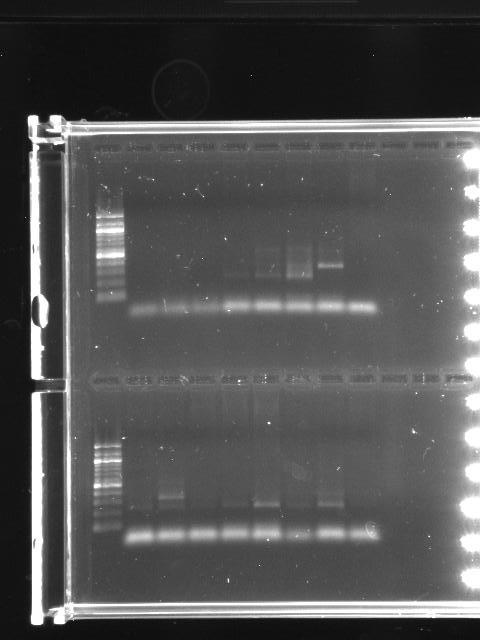

Supplement: Figure S2 — Gel electrophoresis image showing bands present following amplification using nested primers for 10 rounds of PCR on previously amplified PCR products. Top Half: Lane 1: ladder, 2: male positive control, 3: female negative control, 4: water control, 5:#1, 6:#2, 7:#3, 8:#5, 9:#6. Bottom Half: Lane 1: ladder, 2:#7, 3:#8, 4:#9, 5:#10, 6:#11, 7:#12, 8:#13, 9:#20. (JPG) [file pone.0068114.s002.jpg]

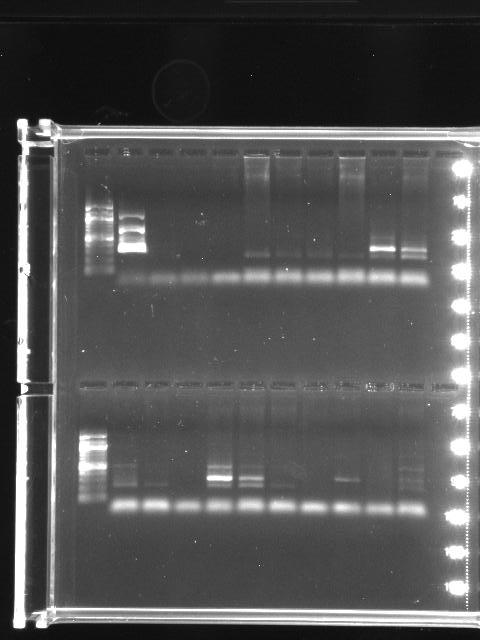

Supplement: Figure S3 — Gel electrophoresis image showing bands present following amplification using nested primers for 10 rounds of PCR on previously amplified PCR products. Top Half: Lane 1:ladder, 2:male positive control, 3:female negative control, 4:water control, 5:#14, 6:#15, 7:#16, 8:#17, 9:#18, 10:#19, 11:#20. Bottom Half: Lane 1:ladder, 2:#24, 3:#25, 4:#26, 5:#27, 6:#28, 7:#29, 8:#30, 9:#31, 10:#32, 11:#33. (JPG) [file pone.0068114.s003.jpg]

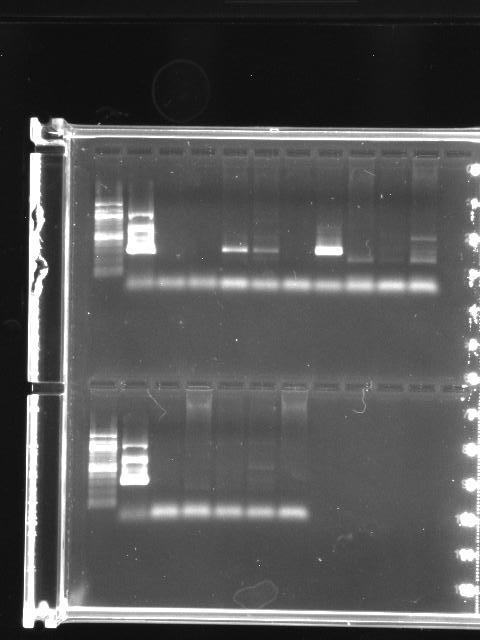

Supplement: Figure S4 — Gel electrophoresis image showing bands present following amplification using nested primers for 10 rounds of PCR on previously amplified PCR products. Top Half: Lane 1:ladder, 2:male positive control, 3:female negative control, 4:water control, 5:#34, 6:#35, 7:#36, 8:#37, 9:#38, 10:#39, 11:#40. Bottom Half: Lane 1: ladder, 2:male positive control, 3:#41, 4:#42, 5:#43, 6:#44, 7:#45. (JPG) [file pone.0068114.s004.jpg]

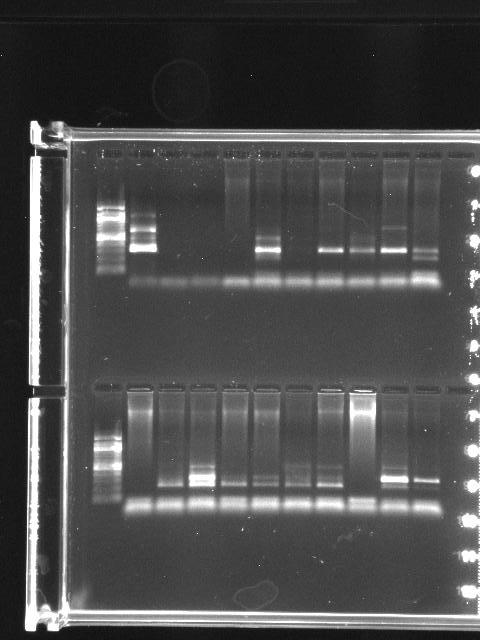

Supplement: Figure S5 — Gel electrophoresis image showing bands present following amplification using nested primers for 10 rounds of PCR on previously amplified PCR products. Top Half: Lane 1:ladder, 2:male positive control, 3:female negative control, 4:water control, 5:#47, 6:#48, 7:#49, 8:#50, 9:#51, 10:#52, 11:#53. Bottom Half: Lane 1:ladder, 2:#54, 3:#55, 4:#56, 5:#57, 6:#58, 7:#59, 8:#60, 9:#61, 10:#62, 11:#63. (JPG) [file pone.0068114.s005.jpg]

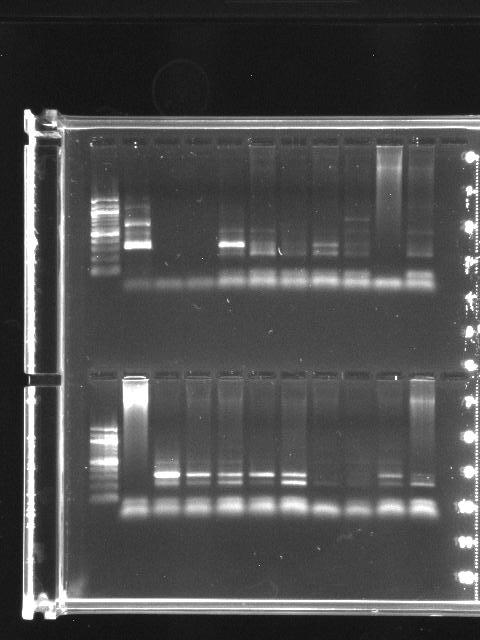

Supplement: Figure S6 — Gel electrophoresis image showing bands present following amplification using nested primers for 10 rounds of PCR on previously amplified PCR products. Top Half: Lane 1: ladder, 2:male positive control, 3:female negative control, 4:water control, 5:#64, 6:#65, 7:#66, 8:#67, 9:#68, 10:#69, 11:#70. Bottom Half: Lane 1: ladder, 2:#71, 3:#72, 4:#73, 5:#74, 6:#75, 7:#76, 8:#77, 9:#78, 10:#79, 11:#80. (JPG) [file pone.0068114.s006.jpg]

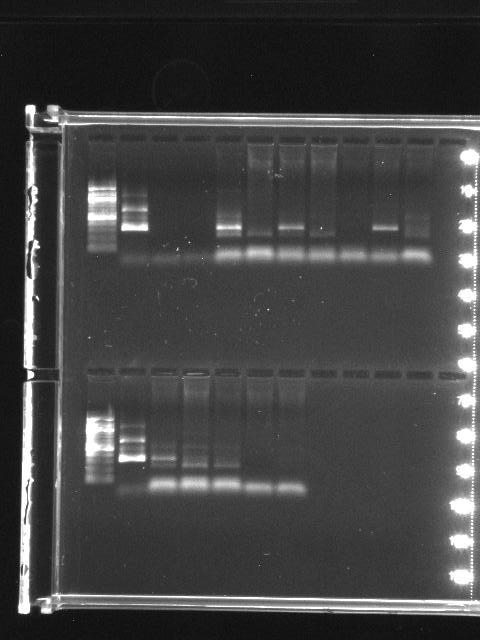

Supplement: Figure S7 — Gel electrophoresis image showing bands present following amplification using nested primers for 10 rounds of PCR on previously amplified PCR products. Top Half: Lane 1: ladder, 2:male positive control, 3:female negative control, 4:water control, 5:#81, 6:#82, 7:#83, 8:#84, 9:#85, 10:#86, 11:#87. Bottom Half: Lane 1: ladder, 2:male positive control, 3:#88, 4:#89, 5:#90, 6:blank, 7:blank. (JPG) [file pone.0068114.s007.jpg]
